# Phospho serine and threonine analysis of normal and mutated granulocyte colony stimulating factor receptors

**DOI:** 10.1038/s41597-019-0015-8

**Published:** 2019-04-09

**Authors:** Pankaj Dwivedi, David E. Muench, Michael Wagner, Mohammad Azam, H. Leighton Grimes, Kenneth D. Greis

**Affiliations:** 1 Department of Cancer Biology, University of Cincinnati, Cincinnati, Ohio 45267 USA; 2 Division of Immunobiology and Center for Systems Immunology, Cincinnati Children’s Hospital Medical Center, Cincinnati, Ohio USA; 3 Division of Biomedical Informatics, Cincinnati Children’s Hospital Medical Center, Cincinnati, Ohio USA; 4 Division of Experimental Hematology and Cancer Biology, Cincinnati Children’s Hospital Medical Center, Cincinnati, Ohio USA

**Keywords:** Acute myeloid leukaemia, Proteomics, Mass spectrometry

## Abstract

Granulocyte colony stimulating factor receptor (G-CSFR) plays an important role in the production of neutrophil granulocytes. Mutated G-CSFRs have been directly associated with two distinct malignant phenotypes in patients, e.g. acute myeloid leukemia (AML) and chronic neutrophilic leukemia (CNL). However, the signaling mechanism of the mutated G-CSFRs is not well understood. Here, we present a comprehensive SILAC-based quantitative phosphoserine and phosphothreonine dataset of the normal and mutated G-CSFRs signaling using the BaF3 cell-line-based *in vitro* model system. High pH reversed phase concatenation and Titanium Dioxide Spin Tip column were utilized to increase the dynamic range and detection of the phosphoproteome of G-CSFRs. The dataset was further analyzed using several computational tools to validate the quality of the dataset. Overall, this dataset is the first global phosphoproteomics analysis of both normal and disease-associated-mutant G-CSFRs. We anticipate that this dataset will have a strong potential to decipher the phospho-signaling differences between the normal and malignant G-CSFR biology with therapeutic implications. The phosphoproteomic dataset is available via the PRIDE partner repository.

## Abstract (summary)

**Table:** 

Design Type(s)	protein physical property analysis objective • replicate design • factorial design
Measurement Type(s)	protein expression profiling
Technology Type(s)	liquid chromatography-tandem mass spectrometry
Factor Type(s)	biological replicate • experimental condition
Sample Characteristic(s)	Mus musculus • BA/F3 cell

Machine-accessible metadata file describing the reported data (ISA-Tab format)

## Background & Summary

Granulocyte colony stimulating factor (G-CSF) also known as colony stimulating factor 3 (CSF3) is the primary ligand for granulocyte colony stimulating factor receptor (G-CSFR)^1,2^. G-CSFR is a transmembrane cytokine receptor consisting of extracellular, transmembrane and intracellular domains^2,3^. There are a number of myeloid disorders that have been related to the mutations in *CSF3R* including Severe Congenital Neutropenia (SCN), Chronic Neutrophilic Leukemia (CNL), Myelodysplastic Syndrome (MDS), Acute Myeloid Leukemia (AML), atypical Chronic Myelogenous Leukemia (aCML)^2–5^. SCN patients treated with G-CSF (in the form of induction therapy) regain sufficient levels of neutrophils to reduce infection related mortality; however, a major concern is the leukemic progression of SCN into MDS or AML^5,6^. Specifically, SCN patients on G-CSF treatment can acquire somatic mutations in *CSF3R*, leading to truncation of the cytoplasmic region of G-CSFR^6^. Other *CSF3R* mutations (specifically the proximal T618I point mutation) are frequently observed *de novo* in CNL that is characterized by the constitutive activation of the receptor, leading to an excess of neutrophils^5^.

**Fig. 1:**
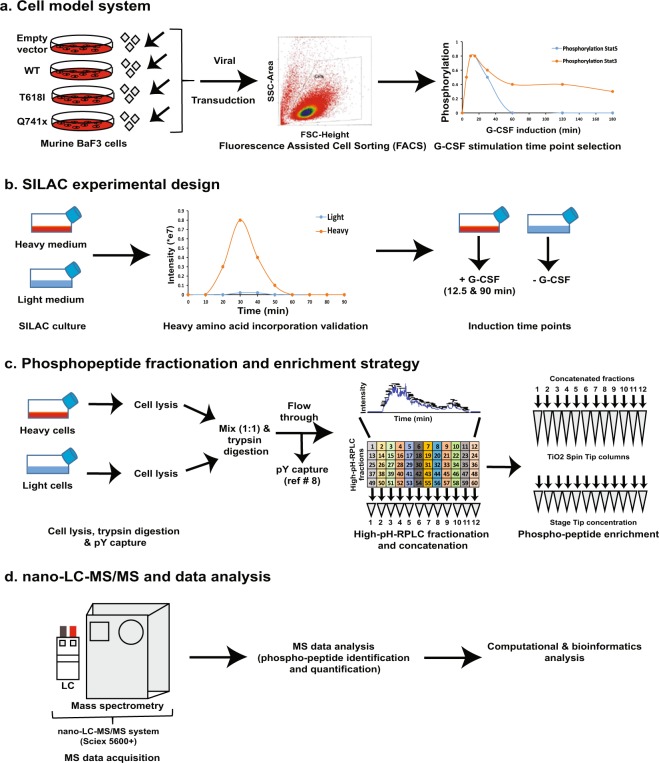
Overall experimental work flow of phospho-serine/threonine analysis. (**a**) Generation and validation of BaF3 expressing WT and mutant G-CSFRs. BaF3 cells were retrovirally transduced for the stable expression of normal and mutated G-CSFRs. The receptor expression was verified by flow cytometry. The transduced cells were further analyzed for the receptor activation kinetics using STATs protein phosphorylation as marker. (**b**) Transduced BaF3 were grown in light and heavy SILAC medium. Heavy amino acid incorporation verification was performed after 5 doubling time and G-CSF induction time point selection was determined based on the previously published literature^8^. (**c**) G-CSF stimulated BaF3 cells were lysed, mixed (equal heavy: light protein amount), digested and desalted prior to phospho-tyrosine (pY) enrichment. The full pY analysis and validation is reported elsewhere^8^. The flow through of pY enrichment was further fractionated/concatenated using high-pH RPLC. The pooled/concatenated fractions were ultimately enriched for phospho-serine/threonine peptides using TiO_2_ Spin Tip columns. (**d**) The enriched phospho-peptides were analyzed by nano-LC-MS/MS on a Sciex 5600+ system. The data analysis was performed with ProteinPilot (Sciex). Additional data analyses were executed with perl and R programming languages. Details of each of these processes in provided in the Methods section.

**Fig. 2:**
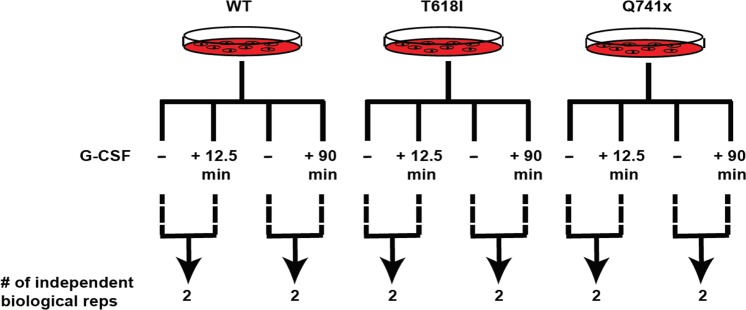
Schematic of the phosphoproteomic experimental design showing the number of 2 independent biological replicates for two time points (early: 12.5 min and late: 90 min) of G-CSF induction for each group (WT, T618I, Q741x). Therefore, the overall study involved 12 independent biological replicates across the study.

Previous studies have shown a differential activation of JAK/STAT pathway downstream of WT and mutated receptor after G-CSF activation^7^. However, the complete signaling biology of G-CSF activated receptor either in the normal or mutated condition is still not known. In the current study, a global profiling of changes in the phosphoproteome from WT, proximal (T618I) and truncated (Q741x) G-CSFR in response with G-CSF in a time dependent manner at 12.5 min (early time point), and 90 min (late time point) post G-CSF induction was performed (Figs 1–2). The workflow included the combination of SILAC labeling, trypsin digestion, pre-fractionation/enrichment to extract phosphotyrosines^8^, high pH RP-chromatography (high-pHRPLC), TiO_2_ enrichment of phospho-serine/threonine (pS/pT), high-resolution nano-LC-MS/MS analysis for phosphorylation changes and bioinformatics analyses (Fig. 1a–d). The selection of induction time points at 12.5 min (early) and 90 min (late—back to baseline) was based on a detailed induction time course to evaluate the phosphorylation dynamics of phospho-Stat5 in the WT and mutated receptor expressing cells^8^. Collectively, more than 10,000 unique phospho peptides (pS/pT) were identified. The phospho-tyrosine dataset for this study included about ~300 sites that have already been published^8^. Here, we present the pS/pT dataset with upward of 1,000 phosphorylation site changes, suggesting a highly dynamic network of cellular signaling that differs between the WT and mutant receptors. Furthermore, the short induction time (particularly for the 12.5 min time point) would strongly suggest that the comparative changes in phosphorylation are due to an increase or decrease in phosphorylation rather than changes in the total amount of a given protein. Given the lack of understating of the phosphorylation dynamics of G-CSFR signaling, this dataset has a great potential to provide the research community with opportunity to further explore normal and variant signaling through the G-CSFR. This dataset may also provide an avenue to understand the mechanism of how the clinically successful induction therapy for SCN patients transition to MDS and AML. Finally, better understanding of the signaling network associated with G-CSFR could also lead to new targets and impact alternative therapeutic strategies for SCN/AML and CNL patients.

## Methods

### Reagents

RPMI was used to culture the BaF3 cells with and without lysine and arginine, fetal bovine serum (FBS), L-glutamine, and antibiotics. Lysine, arginine, FBS, L-glutamine and antibiotics were purchased from Invitrogen (Carlsbad, CA). Heavy amino acids ^13^C_6_-Lysine and ^13^C_6_-Arginine, were obtained from Cambridge Isotope Laboratories (Andover, MA). For proteolytic digestion, TPCK-treated trypsin was used. TPCK-treated Trypsin was purchased from Worthington Biochemical Corp. (Lakewood, NJ). Titanium Dioxide Spin Tip columns were purchased from Thermo Scientific (# 88303). Anti-phospho-Stat3, anti-phospho-Stat5, anti-phospho-Erk antibodies were purchased from Cell Signaling Technology (Danvers, MA). Anti-G-CSFR antibody was purchased from Abcam (Cambridge, MA). All other reagents used in this study were from Fisher Scientific (Pittsburgh, PA). *This section is an expanded version of descriptions in our related manuscript*^8^.

### Immunoblotting and phospho-kinase array

The normal and mutated G-CSF receptors-expressing BaF3 cells were serum starved for 6 hours before G-CSF induction for various time points (ranging from 5 min to 180 min). The cells were washed with PBS followed by lysis in 20 mM Tris-HCl, 150 mM Sodium Chloride, 2 mM EDTA, 1 mM EGTA, Complete Mini Protease Inhibitor Cocktail Tablet (Roche), 10 mM Sodium fluoride, 1 mM Sodium orthovanadate, 1 mM beta-glycerophosphate, 1% NP-40, 1% Tween-20, 10% Glycerol, 2.5 mM Sodium pyrophosphate, 1 mM PMSF. The lysed cellular contents were further sonicated three times at 15 W of 15 sec each. The supernatant was collected post centrifugation at 20,000 × g for 10 min at 4 °C. The protein estimation was performed using 660 nm assay (Thermo Scientific, #22660). 25–30 µg of total lysates were used for each immunoblot analyses. 4–12% Bis Tris gel (Invitrogen, #NP0321) gradient SDS-PAGE gel was used for protein separation. PVDF membrane (Millipore, #IPVH00010) was used for protein transfer using a semi-dry apparatus at 15 V for 30 min. 5% milk solution in TBS-T (0.1% Tween-20, Invitrogen) was used for blocking the PVDF membrane. Phospho-Stat5 (Tyr 694) (C11C5) (#9359), Phospho-Stat3 (Tyr705) (D3A7) (#9145), Phospho-p44/42 MAPK (Erk1/2) (Thr202/Tyr204) (D13.14.4E) (#4370), Stat5 (#9363), Stat3 (#4904), p44/42 MAPK (Erk1/2) (#9102), Actin (#4970) primary antibodies (from Cell Signaling Technologies) & G-CSFR primary antibody (ab#126167, Abcam) were used for the immunoblot analyses. 1:1000 dilutions of primary antibodies were used in 5% BSA solution in TBS-T (0.1% Tween-20). Anti-rabbit and -mouse secondary antibodies from GE Healthcare were used at 1:5000 dilutions in 5% milk solution in TBS-T (0.1% Tween-20). All immunoblots were developed using ChemiDoc^TM^ touch imaging system (Bio-Rad). *This section is an expanded version of descriptions in our related manuscript*^8^.

For phospho kinase array analysis, vendor specific instructions were followed (R&D systems, # ARY003B). Briefly, ~10 million WT and mutant G-CSFRs expressing BaF3 cells were serum starved for 6 hours and stimulated with 40 ng/mL of G-CSF for 12.5 and 90 min. The stimulated cells were washed with 10 mL of ice cold PBS and lysed in 1 mL of lysis buffer provided with kinase array kit. All other wash and reaction steps were done as described in the assay kit using the supplied reagents. After the final wash of the reacted membranes, the chemi-luminescent readout was initiated by incubation with the supplied Chemi Reagent mixture for 1 min then captured using ChemiDoc^TM^ touch imaging system (Bio-Rad). The dot blots generated by the imaging system were further quantified and analyzed using Progenesis SameSpots software. The raw images of the phospho-kinase array study are provided as Supplementary Fig. 1.

### Cell culture and SILAC labeling

BaF3 cell lines stably expressing normal and mutated G-CSFRs, were grown in RPMI medium (Invitrogen, Carlsbad, CA) with 5% fetal bovine serum (FBS), 2mM L-glutamine, 100 U/mL penicillin and 100 µg/mL streptomycin in a humidified incubator at 37 °C with 5.0% CO_2_. For heavy amino acid labeling, the cells were cultured in RPMI with 5% FBS, 2 mM L-glutamine, 100 U/mL penicillin and 100 µg/mL streptomycin, 50 mg/L arginine-^12^C_6_ monohydrochloride and 100 mg/L lysine-^12^C_6_ monohydrochloride (light) or 50 mg/L arginine-^13^C_6_ monohydrochloride and 100 mg/L lysine-^13^C_6_ monohydrochloride (heavy) (Cambridge Isotope Laboratories). The heavy amino acid labeling efficiency was determined after 5 doublings of the cells by removing 1 million actively growing cells from each culture followed by lysis, trypsin digestion and LC-MS analysis. Once the incorporation of heavy amino acids was confirmed to be more than 95%, the cells were collected by centrifugation at 1200 rpm for 5 min. Subsequently, the cells were washed 3 times with PBS, then re-suspended in serum free medium for 6 hours for serum starvation prior to G-CSF stimulation. Cells grown in heavy SILAC medium were stimulated with G-CSF (40 ng/mL) for 12.5 mins and 90 mins at 37 °C and cells grown in light medium were left unstimulated (Fig. 2). Each induction time point experiment was performed as two independent biological replicates (Fig. 2). *This section is an expanded version of descriptions in our related manuscript*^8^.

### Cell lysis and protein digestion

Post G-CSF stimulated BaF3 cells were washed with cold PBS and lysed in 20 mM HEPES pH 8.0, 9 M urea, 1 mM sodium orthovanadate, 2.5 mM sodium pyrophosphate, 1 mM beta-glycerophosphate. The lysed cells were further disrupted by sonication (15 W output with 3 bursts of 15 sec each). During sonication, cell lysates were cooled on ice for 1 min between each burst to avoid any protein denaturation. After sonication, the lysates were cleared by centrifugation at 20,000 × g at 15 °C for 20 min to capture the solubilized protein supernatant. The 660 nM protein assay (Thermo, #22660) was used to measure the total protein amount. 10 mg of light and heavy labeled protein for each pairwise comparison were mixed together before reduction and alkylation steps. The mixed lysate proteins were reduced with DTT at a final concentration of 5 mM at 60 °C for 20 min. Before alkylation, the reduced samples were cooled on ice until it reached room temperature (RT). Alkylation was performed using a final concentration of 10 mM iodoacetamide for 10 min at RT in the dark. Furthermore, the samples were diluted 5X in 20 mM HEPES pH 8.0 to reduce the final urea concentration to less than 2 M for trypsin digestion. TPCK-treated trypsin (Worthington Biochemical Corp) prepared as a stock in 1 mM HCl, was added at a 1:50 (w/w) ratio (trypsin/protein) to the reduced and alkylate lysates for overnight proteolytic digestion at room temperature while rotating. Finally, the peptide mixture was acidified to final concentration of 1% Triflouroacetic acid (TFA) to stop the reaction. *This section is an expanded version of descriptions in our related manuscript*^8^.

### Sep-Pak C18 desalting of lysate peptides

The acidified peptide solution was centrifuged at 1780 × g at RT for 15 min to remove any precipitate. Desalting was done using C-18 Sep-Pak cartridges (Waters, cat# WAT051910). The Sep-Pak columns were washed with a total of 10 mL of 0.1% TFA prior to the peptide loading. The acidified and cleared peptide solution was loaded on Sep-Pak column using gravity flow. Once loading was finished, the column was washed with 10 mL of 0.1% TFA. Peptide elution was performed using a 2 mL of 40% acetonitrile in 0.1% TFA solution. The elution step was repeated three times and all the eluate were combined at the end. The eluted peptides were lyophilized for at least 3 days, reconstituted in 1.4 mL of immuno affinity buffer (20 mM Tris-HCl, 10 mM Sodium Phosphate, 50 mM Sodium Chloride, pH 7.4) and subjected to phosphotyrosine capture using pY1000 cartridges from Cell Signaling Technologies (#8803) using the vendor supplied instructions. The flow through and wash from the phospho-tyrosine enrichment was collected, frozen, lyophilized in a SpeedVac and stored at −80 °C until further analysis. *This section is an expanded version of descriptions in our related manuscript*^8^.

### High-pH reversed-phase liquid chromatography (high-pHRPLC) and TiO_2_-based phosphopeptide enrichment

Peptides were fractionated by high pH reversed-phase liquid chromatography. Briefly, the dried flow through from the pY1000 cartridge was reconstituted in 1 mL of high-pHRPLC solvent (10% Ammonium Formate in water pH 10) and fractionated by high-pHRPLC chromatography on a XBridge C18, 5 µm, 250 × 4.6 mm column (Waters Corporation, Milford, MA) by employing an increasing gradient of solvent B (10% Ammonium Formate in 90% Acetonitrile pH 10) on an Ultimate Plus by LC Packings HPLC with a flow rate of 250 µL/min. 1.5 min fractions (375 µL) over 90 minutes were collected for each separation for a total of 60 fractions. The high-pH fractions were then concatenated by pooling every 12^th^ fraction (1, 13, 25, 37, 49; then 2, 14, 26, 38, 50;…12, 24, 36, 48, 60) to generate 12 pooled fractions (Fig. 1c). Due to the limited capacity of the XBridge column, each 20 mg sample was split in half and run as two subsequent separations and combine with the corresponding concatenated fractions such that the final output of the high-pHRPLC for each sample set was 12 pooled fractions that were lyophilized in a SpeedVac.

For phospho-peptides enrichment, each concatenated fraction was subjected to a TiO_2_ spin column following the vendors instructions (Thermo Scientific, # 88301). Briefly, each spin column was first washed with 10 µL of buffer A (80% acetonitrile in water) and buffer B (40% lactic acid in buffer A) each. The dried peptide fractions were suspended in 20 µL of buffer B. The peptide solution was applied to the spin columns and centrifuges at 1000 × g for 2 min. The pass through from the column was applied again to the column to increase the enrichment efficiency of phosphopeptides. Next, the column was washed with 20 µL each of buffer B and A respectively. The enriched phosphopeptides were eluted with 50 µL of elution buffer (30% ammonium hydroxide solution in water) twice. The eluted enriched phosphopeptides were dried using speed vac and stored at −80 °C before further analysis as described previously^9^.

### Concentration of enriched phosphopeptides for LC-MS using StageTips

The enriched phosphopeptides were desalted/concentrated prior to LC-MS/MS analysis using custom made StageTips in the lab^10^. Empore High Performance Extraction C18 disks (AHO-2540 Phenomenex) were punched twice with P200 pipette tip and the N_2_ gas pressure flow was used to transfer the punched C18 material into a P20 pipette tip (StageTip). Each Tip was washed with 40 µl of 50% acetonitrile in 0.1% TFA solution by spinning at 2000 × g for 5 min. Next, the membrane was prepared for peptide binding by washing with 40 µl of 0.1% TFA solution at 2000 × g for 5 min. The enriched phospho-serine/threonine peptides (dried form) were reconstituted in 40 µl of 0.1% TFA prior to the loading/binding on to the StageTip. The binding step was performed at 2000 × g for 10 min. The flow through of the load step was further applied to the StageTip columns (two sequential binding steps total). Post binding, the columns were washed with 40 µl of 0.1% TFA twice. The elution was performed with 40 µl of 40% acetonitrile twice. The eluate was dried using speed vac and stored at −80 °C before LC-MS/MS analysis.

### Nano-LC-MS/MS Analysis

Nano-LC-MS/MS analyses on the phosphopeptide fractions were performed using a TripleTOF 5600+ MS (Sciex, Toronto, ON, Canada). Mass spectrometry was coupled with an Eksigent (Dublin, CA) nanoLC.ultra-system. Concentrated dried phosphopeptides were resolubilized in 0.1% formic acid in water (v/v) prior to the loading on IntegraFrit trap column (outer diameter of 360 µm, inner diameter of 100 µm, and 25 µm packed bed) from New Objective (Woburn, MA). The reconstituted peptide solution was loaded onto the trap column at 2 µl/min in 0.1 formic acid in water (v/v) for 10 min to desalt and concentrate the sample. For the analytical/chromatographic separation of phosphopeptides, the trap column was switched to align with the nanoLC separation column, an Acclaim PepMap 100 (inner diameter 75 µm, length 15 cm, C18 particle size of 3 µm, 100 A of pore size) from Dionex-Thermo Fisher Scientific (Sunnyvale, CA). The peptide elution step was achieved using a varying mobile phase gradient from 95% phase A (0.1% formic acid in water, v/v) to 40% phase B (0.1% formic acid in acetonitrile, v/v) for 70 min, from 40% phase B to 85% phase B for 5 min, next keeping the same mobile phase composition for 5 min at 300 nL/min as described previously^9^.

Positive ion mode was used to operate the mass spectrometer using 4303 cycles for 90 min. 0.25 sec accumulation time and 350–1600 m/z window were used in each TOF-MS cycle. 20 information dependent acquisition (IDA) MS/MS-scans on the most intense candidate ions which had a minimum of 150 counts, were collected per cycle. An accumulation time of 0.05 sec and a mass tolerance of 100 ppm were used for the product ion scan.

*This section is an expanded version of descriptions in our related manuscript*^8^.

### Mass spectrometric data analysis

The data generated by nano-LC-MS/MS (.wiff files) from the enriched phosphopeptides were further analyzed for protein identification and quantification using Protein Pilot software (version 5.0, revision 4769). Protein Pilot utilized the Paragon algorithm and was searched against a UniProt database of *Mus musculus* protein sequences. Each phospho-enriched raw MS data was processed using the SILAC specific settings in Protein Pilot: sample type (SILAC (Lys + 8, Arg + 10)), Cys Alkylation (Iodoacetamide), Digestion (Trypsin), instrument (Triple TOF 5600), and Special Factors (Phospho-emphasis). False Discovery Rate (FDR) was set as 0.05 with through ID as search effort. A combined search run was performed for the 12 MS runs (phospho-enriched samples from 12 high pH concatenated fractions) for phospho-serine/threonine peptides. The search results were generated as.group files in an excel spreadsheet as a peptide summary report. A minimum of 95% confidence in identity (calculated by probability algorithm of Protein Pilot software) was used as a cut-off for phosphopeptides identification. Furthermore, sequence, modification, mass-to-charge ratio (m/z value), and charge (z) were as selection parameters for phosphopeptide data filtration as published previously^9^.

### Phosphoproteomic data processing using perl

A custom perl script was used to pre-process and normalize the phospho-proteomic dataset. This script utilized the raw data (search results generated by Protein Pilot as.group files in a excel spreadsheet as peptide summary report), filtered out identifications at less than a 95% confidence level based on the Protein Pilot processing output, and normalized the peptides based on the median-normalized ratios of peptide intensities for receptor-activated vs. non-activated states (G-CSF treated vs. non-G-CSF treated). In cases where the same peptide was identified multiple times, the intensity data was averaged for that peptide. If a peptide was consistently detected in only one of the two (heavy or light) SILAC channels, log ratios of heavy to light peptide intensities were artificially set to plus or minus infinity. Furthermore, peptide ratios were normalized within replicates to have a median of 0 (i.e., no change between activated and non-activated states). Pearson correlation coefficients (r) between replicates were calculated to access the biological/technical reproducibility; these were shown to be very high at greater than 0.85 for all datasets. To understand the inter-relationship between the G-CSFR phosphorylation landscape between WT and mutant signaling, an unsupervised heatmap and clustering analysis was performed using R. The clustering analysis showed a reproducibility of each independent replicated by displaying their close cluster (Fig. 5a). For the final heatmap and clustering analysis (Fig. 5b), only those phospho-sites which were detected in both biological replicates were used. *This section is an expanded version of descriptions in our related manuscript*^8^.

## Data Records

The raw data (.wiff files), group searched files (Protein Pilot.group files), and quant files [log2 (heavy/light) expression file: excel file format] resulting from phospho-Serine/Threonine analysis, have been deposited to the ProteomeXchange Consortium (http://proteomecentral.proteomexhange.org) via the PRIDE partner repository^1^. The extracted phospho-tyrosine dataset of the experimental work-flow involved here has been submitted independently and published^8^. The raw data (.wiff files), group searched files (Protein Pilot.group files), and quant files [log2 (heavy/light) expression file: excel file format] of the phospho-tyrosine data have been deposited to the ProteomeXchange Consortium (http://proteomecentral.proteomexhange.org) via the PRIDE partner repository^11^.

## Technical Validation

### Sample preparation and quality control for G-CSFR signaling biology in the model system

Given that a global phosphoproteomics study needs large amount of starting material, a robust system was required which could be easily grown with high protein yield. The BaF3 cell system has been used as a workhorse to study G-CSFR signaling biology previously^2–6^. Furthermore, they are easy to grow and endogenous levels of G-CSFR are not detectible by flow cytometry^8,12^, but they can be programmed to express receptors using lenti- and retroviral vectors^8,12^. Based on all these properties, BaF3 cells were chosen to make an *in vitro* model system expressing empty vector, WT and mutated G-CSFRs (T618I and Q741x) respectively^8^. A retroviral transduction was performed with BaF3 cells and G-CSFRs expressing cells were further sorted by flow cytometry to isolate cell populations expressing physiologically relevant levels of WT or mutant receptors as illustrated in Fig. 1a. To ensure minimal variations due to the cellular background, all 4 receptor expressing cell types (empty vector, WT, T618I and Q741x) were generated by parallel transduction into a single BaF3 cell background as detailed elsewhere^8^.

**Fig. 3:**
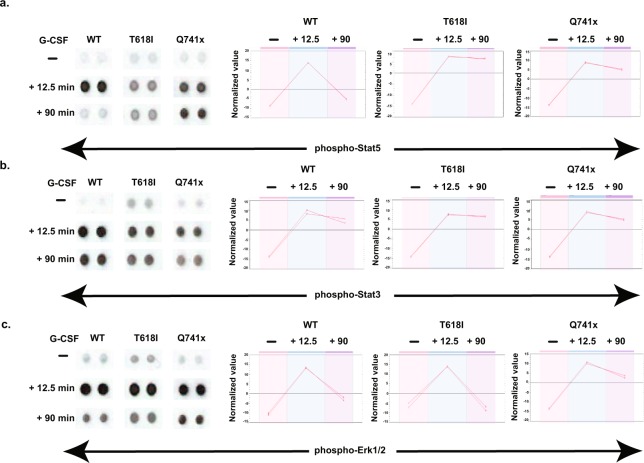
Phospho kinase arrays were used on the transduced BaF3 cells to validate the canonical JAK/STAT and MAPK/ERK signaling pathways downstream of the activated and non-activated receptors all as described in the Methods section. (**a**) BaF3 cells expressing normal and mutated G-CSFRs were serum starved for 6 hours and induced with 40 ng/mL G-CSF for 12.5 min and 90 min before the cells were lysed and the array membranes were treated with the lysates. The lysates treated membranes were exposed to primary and secondary antibody cocktail provided by the vendor and signal was measured with ChemiDoc^TM^ touch imaging system for phospho-Stat5, phospho-Stat3, and phospho-Erk1/2 (panels: a–c respectively). The full images of the membrane blots are also provided as Supplementary Fig. 1. The quantitative data analysis was performed using Progenesis SameSpots software with the relative spot volumes plotted in the right panels for the indicated phosphorylation sites.

Based on patient derived samples, an abnormal activation of STAT5, STAT3 and ERK 1/2 have been reported with mutations in G-CSFRs when compared with the WT^5,6^. This includes a constitutive level of STAT3, STAT5 and ERK 1/2 activation in cells from patients with T618I mutation, and a prolonged phosphorylation of these regulatory proteins in patients with the Q741x truncation mutation^5,6^. To confirm whether our *in vitro* system using receptors expressed on BaF3 cells can recapitulate similar signaling biology, the following validation experiments were performed. First, we performed an immunoblot analysis of a time course of G-CSFR activation in the BaF3 model system clearly shows that the T618I mutation maintains a constitutive level of activation even after 6 hours of serum starvation, while receptor activation remains quiescent in WT and Q741x expressing cells^8^. Secondly, the sustained or elevated activation of Stat3, Stat5 and Erk1/2 in the truncation mutation (Q741x) compared to the WT goes well beyond 2 hours as is reported for patient derived samples^5,6^. Finally, evaluation of the G-CSFR activation dynamics in the BaF3 model system using a phospho-kinase array method (Fig. 3 and Supplementary Fig. 1), also recapitulated the phosphorylated dynamics as reported for patient derived mutations in the G-CSFR. Thus collectively, by carefully designing the BaF3 system to express the WT, T618I or Q741x forms of G-CSFR at equal levels, a valid model system has been established that can be scaled up to levels sufficient for investigating the phosphorylation dynamics in the normal and disease-associated receptor mutations.

### Quality check of phosphopropteomics data analysis

**Fig. 4:**
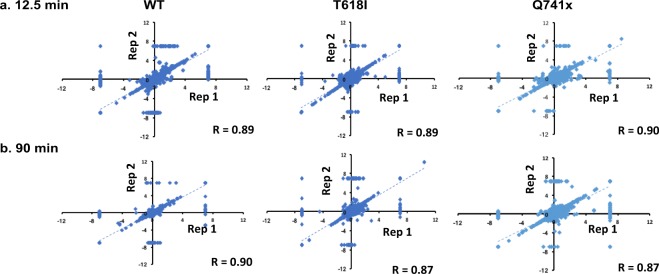
Pearson’s Correlation coefficients (r) values were calculated to verify the biological reproducibility between independent replicates in each group (WT and mutants). Each data point represents a single phosphorylation site that was identified and quantified versus the non-stimulated control sample. The extent to which the relative quantitation of the biological replicates (rep 1, rep 2) are reproducible, can be determined by how closely the replicate values produce a correlation closest to 1. **(a)** At both early (12.5 min) and (**b**) the late time (at 90 min) points after G-CSF stimulation, the reproducibility observed between the replicates were all correlated at >0.85. Axis for each replicate are on a log base 2 scale.

**Fig. 5:**
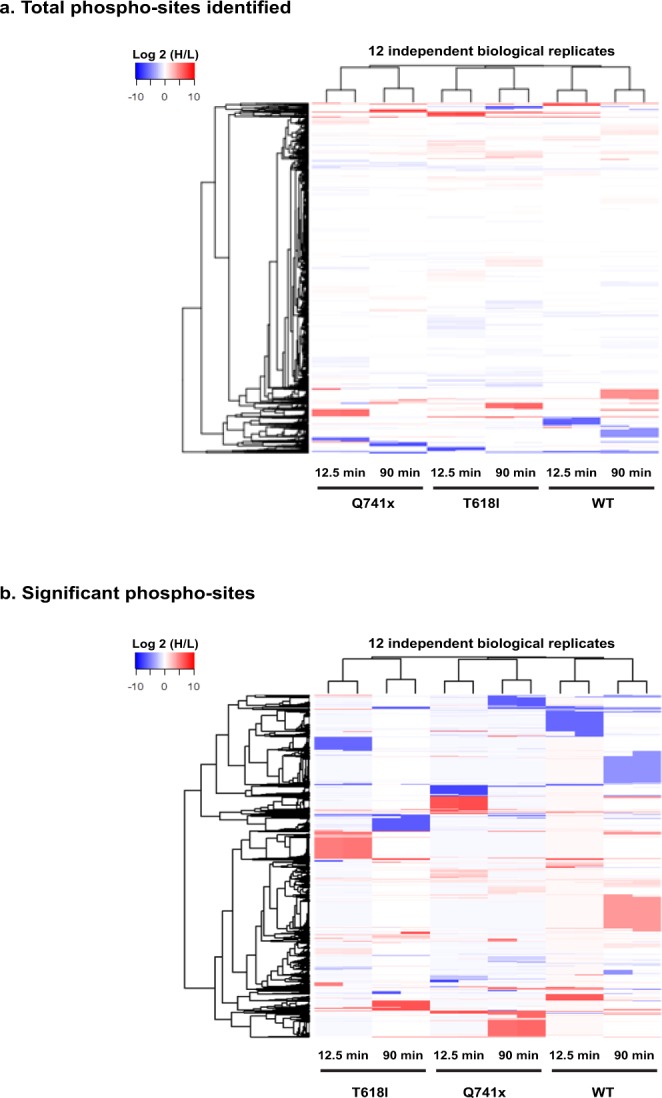
Global quantitative phospho-serine and phospho-threonine analyses display a differential phosphorylation pattern between normal and mutated G-CSFR expressing cells after induction with G-CSF. (**a**) R-based unsupervised hierarchical clustering heat map analyses were performed using log2 transformed heavy to light phospho-serine and phospho-threonine peptide ratios from >10,000 phosphorylation sites. The color key denotes red as up-regulated and blue color as down-regulated sites respectively as compared to pre induction with G-CSF. (**b**) The data were further filtered to include a strict stringency of the overall global dataset where only those phosphopeptides detected in both replicates (no imputation) and demonstrating ≥log2 (1.5) or ≤log2 (1.5) heavy/light ratio were filtered for a heatmap and clustering analysis. This included a total of ~ 1000 phosphorylation sites.

Each experimental condition of G-CSF induction was performed in two independent biological replicates. To measure the correlation between experimental findings, expression data were plotted using Pearson correlation analysis (Fig. 4). This analysis was done between each replicate and each time point (e.g., two independent biological replicate of WT group at 12.5 min—an early time point of G-CSF induction). The Pearson correlation analysis between each replicate showed a high reproducibility with coefficient (r) values all greater than 0.85 (Fig. 4). However, we did observe some outliers in the correlation analyses as depicted in Fig. 4. Most of these outliers stem from missing values among the replicates but even with these included, the correlations are still very high. Additionally, when evaluating the full dataset using unbiased heatmap as well as clustering analysis, each pairwise replicate clustered most closely to its biological replicate thus further validating the overall reproducibility of the dataset (Fig. 5a,b). We further filtered all identified phosphopeptides (>10,000) by removal of missing values (no imputation) and selection of only plus or minus log2 (1.5) (Heavy/Light) ratio to enriched the significant sites and their clustering pattern across G-CSFR phosphoproteome landscape (Fig. 5b). Collectively, upward of 1,000 phosphorylation site changes were observed after stringent filtering of the all identified phosphorylation sites.

In conclusion, the dataset presented passes both on the biological relevance of the model system (compared to known signaling dynamics from patient derived samples) and the reproducibility of the biological replicates for the SILAC, phospho-enrichment workflow. Hence the depth of the dataset with over 10,000 pS/pT phosphorylation sites detected and over 1,000 phosphorylation sites that showed at least a 50% change in stimulated versus non-stimulated conditions, provides a valuable dataset to be shared with the research community. An initial pass at cluster analysis begins to show unique clusters of phosphorylation among the variant group that will need to be exploited further (Fig. 5). We expect that these data can be mined further to advance the understanding of the dynamics of normal and variant G-CSFR signaling in disease.

## Supplementary Information

### ISA-Tab metadata file

Download metadata file

### Supplementary Information

Supplementary Figure 1

Supplementary File 1

## Data Availability

A custom code was written in perl programming language to parse and filter the phosphoproteomics data automatically, presented in this study. The code is provided as supplementary material (Supplementary File 1).
