# Supplementary material for: Phospho serine and threonine analysis of normal and mutated granulocyte colony stimulating factor receptors
Source: Sci Data. 2019 Apr 9;6:21. doi: 10.1038/s41597-019-0015-8 (PMC6480977; doi:10.1038/s41597-019-0015-8)
Supplement: Supplementary file 2 — Supplementary Figure 1 [file 41597_2019_15_MOESM2_ESM.pdf]

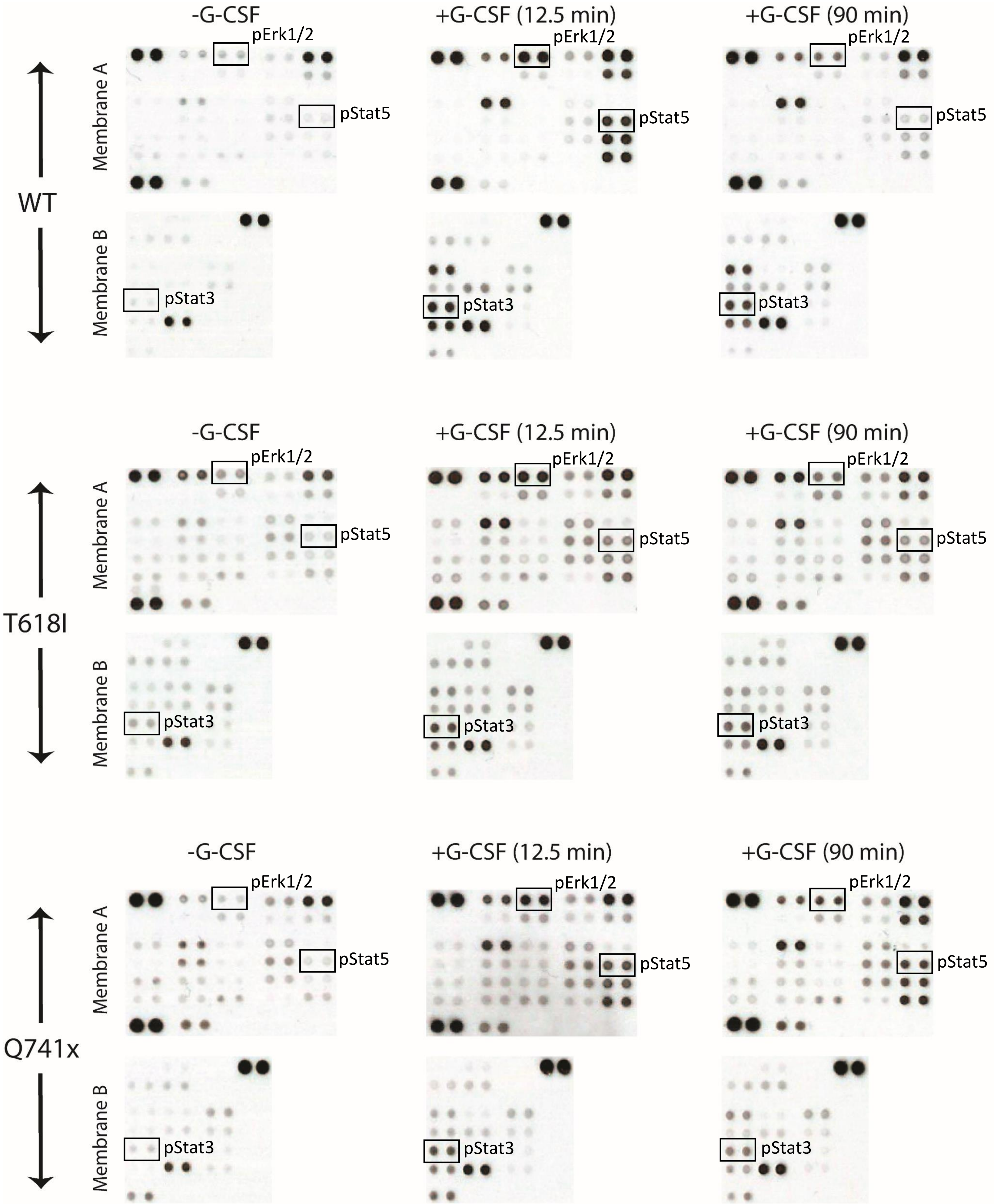

Supplementary Figure 1. Full Human Phospho-Kinase Array membrane profiles for each growth condition and time point in the phosphoproteomics study. R&D systems arrays were used as described under the Immunoblotting and phospho-kinase array heading in the Methods section. The boxed regions for the indicated phosphoproteins were copied to create the summary profiles in Figure 3. Full details of the kinase array lay out and specific phosphorylation sites for each spot are available in the product description for R&D catalog number ARY003B and through the following link (https://resources.rndsystems.com/pdfs/datasheets/ary003b.pdf).
