# Supplementary material for: Phospho serine and threonine analysis of normal and mutated granulocyte colony stimulating factor receptors
Source: Sci Data. 2019 Apr 9;6:21. doi: 10.1038/s41597-019-0015-8 (PMC6480977; doi:10.1038/s41597-019-0015-8)
Supplement: Supplementary file 3 — Supplementary File 1 [file 41597_2019_15_MOESM3_ESM.pdf]

```
#!/usr/bin/perl

use strict;
use Statistics::Basic qw(:all);
use Getopt::Long;
use Pod::Usage;

#####################################################################################
#############
#
# perl script to read and normalize quantitative proteomics data
# Pankaj Dviwedi and Michael Wagner, March 2017
#
# median-normalizes logH/L values (shifts median to 0)
# if $impute = 1, imputes logH/L to 0 where either H or L are 0, or protein is not detected
#
#####################################################################################
#############


# parameters that can, in theory, be changed... but change with care

my $modification = "Phospho"; # this is the identifying string for the modification of interest -
changing this will require more work
my $confidence_threshold = 95;

my $file_extension = ".txt";
my $separator = "\t"; #separator in data files, needs to change if format were to change to, say,
csv

#my $data_folder = "/Users/wagwb8/Pankaj/Phospho-Serine-Threonine/";
#my $file_extension = ".csv";
#my $separator = ",";

my $impute = 0; # 0 = do not impute (default), 1 = perform imputation (set missing log ratios to
median, which after normalization is 0.

GetOptions(#'modification=s' => \$modification,
		'confidence_threshold=i' => \$confidence_threshold,
		'data_folder=s' => \$data_folder,
		'file_extension=s' => \$file_extension,
		'separator=s' => \$separator,
		'impute=i' => \$impute) or pod2usage(1);
```

```
my $results_folder = $data_folder."results_n_thresh=1/";

###############################################################################
#############

# grab all files in data folder that fit the pattern
my @filepaths = glob $data_folder."*".$file_extension;

print join("\n", @filepaths);

###############################################################################
##############

my %unique_sequences;
my %min_pos_L;
my %min_pos_H;
my %Accessions;
my %Names;
my %TheorMW;
my %Sum_Area_L;
my %Sum_Area_H;
my %n_L;
my %n_H;
my %n;
my %n_only_L_pos;
my %n_only_H_pos;
my $n_thresh = 1; # minimum number of times that a peptide needs to show up with L or H = 0
to be counted as real

my %both_H_L_pos;
my %only_H_pos;
my %only_L_pos;

my %median_H_div_L;
my %log_H_div_L;

my %Count;

my %data;

my $counter;

my %line_count;
```

```
my $OUTPUT_AUTOFLUSH = 0;
$| = 1;

my @exp_names;

print "\n\n";

##############################################################################
############
# parse experiment names from file names
##############################################################################
############

foreach my $filepath (@filepaths)
{
  if ($filepath =~ /^.*\/(.*)$file_extension$/)
  {
     @exp_names = (@exp_names, $1);
     print "Found experiment named $1\n";
  }
}

##############################################################################
#############

# now go through files AGAIN and summarize H and L for high-confidence peptides with mod of
interest

print "\n\nComputing average Area_L and Area_H for all unique peptides\n";
print "+++++++++++++++++++++++++++++++++++++++++++++++++++++++++++++++++\n";
print "+++++++++++++++++++++++++++++++++++++++++++++++++++++++++++++++++\n\n";


foreach my $exp_name (@exp_names)
{

   #print "Experiment:\t".$exp_name."\n";

   $counter = 0;

   my %local_unique_sequences;

   my $filepath = $data_folder.$exp_name.$file_extension;
```

```
#print "Opening $filepath.....";

open (INPUTFILE, $filepath) or die "Cannot open input file $filepath\n";
open (LOGFILE, ">$filepath.log") or die "Cannot open logfile $filepath\n";

#read first line in as header line
my $headline = <INPUTFILE>;

chomp($headline);
my @header = split $separator, $headline;

#replace white space in header line names to enable referencing in hashes
s/ /_/g for @header;

        my $prev_unique_seq = "";
        my $unique_seq = "";
        my $both_H_L_pos = 0;
        my $only_L_pos = 0;
        my $only_H_pos = 0;

while (<INPUTFILE>)
{
   chomp;

        my %dataline;

        @dataline{@header} = split $separator;

        if (($dataline{Conf} >= $confidence_threshold)
           && ($dataline{ProteinModifications} =~ /$modification/)
           && ($dataline{Area_L} || $dataline{Area_H}))
        {

           $unique_seq = "$dataline{Sequence}\t".$dataline{ProteinModifications};


           #print "Found data line for $unique_seq\n";

           $Accessions{$unique_seq} = $dataline{Accessions};
           $Names{$unique_seq}      = $dataline{Names};
           $TheorMW{$unique_seq}    = $dataline{Theor_MW};

           $local_unique_sequences{$unique_seq}++; #unique phospho-site sequences per file
```

```
		$unique_sequences{$unique_seq}++; # global master list of unique phospho-site
sequences

		$Count{$exp_name}{$unique_seq}++; #count of how often this unique sequence
appears in this file

		if (($dataline{Area_L} > 0) && ($dataline{Area_H} > 0)) # only use data where both H
and L are present
		{
			$both_H_L_pos{$exp_name}{$unique_seq} = 1;

			$Sum_Area_L{$exp_name}{$unique_seq} += $dataline{Area_L};
			$Sum_Area_H{$exp_name}{$unique_seq} += $dataline{Area_H};

			$n{$exp_name}{$unique_seq} += 1;
		}
		elsif(($dataline{Area_L} > 0) && ($dataline{Area_H} == 0))
		{
			$only_L_pos{$exp_name}{$unique_seq} = 1;
			$n_only_L_pos{$exp_name}{$unique_seq} += 1;
		}
		elsif(($dataline{Area_H} > 0) && ($dataline{Area_L} == 0))
		{
			$only_H_pos{$exp_name}{$unique_seq} = 1;
			$n_only_H_pos{$exp_name}{$unique_seq} += 1;
		}

		print LOGFILE "Found data line for
".$unique_seq."\t".$dataline{Area_H}."\t".$dataline{Area_L}."\tn =
".$n{$exp_name}{$unique_seq}."\tcode=";
		if ($both_H_L_pos{$exp_name}{$unique_seq})
		{
			print LOGFILE "B";
		}
		else
		{
			print LOGFILE "b";
		}

		if ($only_L_pos{$exp_name}{$unique_seq})
		{
			print LOGFILE "L";
		}
		else
```

```
            {
                print LOGFILE "l";
            }
            if ($only_H_pos{$exp_name}{$unique_seq})
            {
                print LOGFILE "H";
            }
            else
            {
                print LOGFILE "h";
            }
            print LOGFILE "\n";

            $counter++;
        }
    }

    close INPUTFILE;
    close LOGFILE;

    printf "Found a total of $counter high-confidence $modification-peptides in $exp_name, ".
        "%d of which are unique\n", scalar keys %local_unique_sequences;

    #print join ("\n", sort keys %local_unique_sequences);

}

printf "Total number of unique high-confidence (p >= $confidence_threshold) ".
    "$modification-peptides in all %d files: %d\n\n\n", scalar @filepaths, scalar keys
%unique_sequences;


####################################################################################
#############
#
# Go though all experiments' processed summary files, compute log ratios and medians thereof
#
####################################################################################
#############

my %median_log_H_div_L;

foreach my $exp_name (@exp_names)
{
```

```
    open (OUTPUTFILE, ">$results_folder$exp_name.processed.txt")
            or die "Cannot write to $results_folder.$exp_name.processed.txt\n";

    print "Writing to file $results_folder$exp_name.processed.txt\n";

    # print header line

    print OUTPUTFILE
"Accessions\tNames\tSequence\tProteinModifications\tTheorMW\tnum_peptides with heavy
or light peak\tAvg_Area_L\tAvg_Area_H\tlog_H_div_L\n";

    my @log_H_div_L = ();

    foreach my $seq (sort keys %unique_sequences)
    {
            if ($Count{$exp_name}{$seq})
            {
               #print $filepath."\t".$seq."\t".$Count{$filepath}{$seq}."\n";

               if ($both_H_L_pos{$exp_name}{$seq})
               {
                       my $Avg_L       =   $Sum_Area_L{$exp_name}{$seq}/$n{$exp_name}{$seq} if
$n{$exp_name}{$seq};
                       my $Avg_H       =   $Sum_Area_H{$exp_name}{$seq}/$n{$exp_name}{$seq} if
$n{$exp_name}{$seq};

                       my $Avg_H_div_L =   $Avg_H / $Avg_L;
                       printf OUTPUTFILE "%s\t%s\t%s\t%s\t%d\t%.2f\t%.2f\t%.5f\n",
                          $Accessions{$seq},$Names{$seq},$seq,$TheorMW{$seq},
                          $Count{$exp_name}{$seq},$Avg_L, $Avg_H, log($Avg_H_div_L)/log(2);
                       push @log_H_div_L, log($Avg_H_div_L)/log(2);
               }
               elsif ($only_H_pos{$exp_name}{$seq} && !$only_L_pos{$exp_name}{$seq} &&
($n_only_H_pos{$exp_name}{$seq} >= $n_thresh))
               {
                       printf OUTPUTFILE "%s\t%s\t%s\t%s\t%d\t0\tinf\tinfinity\n",
                          $Accessions{$seq},$Names{$seq},$seq,$TheorMW{$seq},
                          $Count{$exp_name}{$seq};
               }
               elsif ($only_L_pos{$exp_name}{$seq} && !$only_H_pos{$exp_name}{$seq} &&
($n_only_L_pos{$exp_name}{$seq} >= $n_thresh))
               {
                       printf OUTPUTFILE "%s\t%s\t%s\t%s\t%d\tinf\t0\tneg_infinity\n",
                          $Accessions{$seq},$Names{$seq},$seq,$TheorMW{$seq},
```

```
                        $Count{$exp_name}{$seq};
                }
                else
                {
                        printf OUTPUTFILE "%s\t%s\t%s\t%s\tND\tND\tND\tND\n",
$Accessions{$seq},$Names{$seq},$seq,$TheorMW{$seq};
                }
        }
        else
        {
                printf OUTPUTFILE "%s\t%s\t%s\t%s\tND\tND\tND\tND\n",
$Accessions{$seq},$Names{$seq},$seq,$TheorMW{$seq};
        }
    }
    close (OUTPUTFILE);

    #print join("\t", @log_H_div_L)."\n";
    $median_log_H_div_L{$exp_name} = median(@log_H_div_L);
}

foreach (@exp_names)
{
    print "$_ -> median log H/L = ".$median_log_H_div_L{$_}."\n";
}

####################################################################################
#############
#
# Finally, go through all processed files and write final summary table of median-normalized
values.
# Impute missing values to zero if desired
#
####################################################################################
#############

my @protein_phosphos;

my %count;

my %fhs; # hash of file handles

foreach my $exp_name (@exp_names)
{
    my $f = "$results_folder$exp_name.processed.txt";
```

```
    open (my $fh, '<', $f) or die "Unable to open $f: $!";
    $fhs{$exp_name} = $fh;
}

my $outfilename = $results_folder."logH_div_L_summary.impute=".$impute.".txt";

open (OUTPUTFILE, ">$outfilename") or
    die "Cannot open and write to $outfilename\n";

print "Writing to results file $outfilename\n";

print OUTPUTFILE "Accessions\tSequence\tPhosphorylation
Site\tAccessions\tGene\tSequence\tSite\tmodification_types\t".
    join("\t", @exp_names)."\tNumber of Nonzeros\n";

my @header_lines;

foreach my $exp_name (@exp_names)
{
    my $head = readline $fhs{$exp_name};

    chomp($head);

    #print $head;

    @header_lines = (@header_lines, $head);
}

my @headers = split "\t", $header_lines[0];

my $counter = 0;

while (! eof ($fhs{$exp_names[0]}))
{
    $counter++;

    my @data_lines;

    foreach my $exp_name (@exp_names)
    {
            my $data = readline $fhs{$exp_name};

            chomp($data);
```

```
    @data_lines = (@data_lines, $data);
}
```
